# Investigating the Phenotypic Plasticity of the Invasive Weed *Trianthema portulacastrum* L.

**DOI:** 10.3390/plants11010077

**Published:** 2021-12-27

**Authors:** Marwa A. Fakhr, Yasser S. A. Mazrou, Faten Y. Ellmouni, AlBaraa ElSaied, Mohamed Elhady, Amr Elkelish, Iman H. Nour

**Affiliations:** 1 Botany Department, Faculty of Science, Fayoum University, Fayoum 63514, Egypt; 2 Plant Protection and Biomolecular Diagnosis Department, Arid Lands Cultivation Research Institute, City of Scientific Research and Technological Applications (SRTA-City), New Borg El-Arab City, Alexandria 21934, Egypt; 3 Business Administration Department, Community College, King Khalid University, Guraiger, Abha 62529, Saudi Arabia; ymazrou@kku.edu.sa; 4 Faculty of Agriculture, Tanta University, Tanta 31512, Egypt; 5 Botany and Microbiology Department, Faculty of Science, Al-Azhar University, Cairo 11651, Egypt; albraa.mahmoud@azhar.edu.eg (A.E.); Elhadymohamed566@Yahoo.com (M.E.); 6 Botany Department, Faculty of Science, Suez Canal University, Ismailia 41522, Egypt; amr.elkelish@science.suez.edu.eg; 7 Botany and Microbiology Department, Faculty of Science, Alexandria University, Alexandria 21511, Egypt

**Keywords:** cluster analysis, invasive weed, leaf micromorphology, morphometric analysis, phenotypic plasticity, seed micromorphology, *Trianthema portulacastrum*

## Abstract

Phenotypic plasticity is frequently highlighted as a key factor in plant invasiveness, as it enables invasive species to adapt to diverse, complicated habitats. *Trianthema portulacastrum* is one of the most common aggressive species that threaten different crops around the world. Phenotypic plasticity in *T. portulacastrum* was investigated by comparing variation in germination, vegetative macromorphology, photosynthetic pigments, stomatal complexes, and seed micromorphological traits of 35 samples collected from 35 different localities. One-way cluster analysis and principal component analysis (PCA) were used to classify samples into homogeneous groups based on the measured traits. Pairwise statistical comparisons were conducted between the three resulting groups. The phenotypic plasticity index (PI) was calculated and compared among different groups of characters. Results showed that photosynthetic pigments and macromorphological characteristics had the highest PI, followed by seed micromorphology, and then stomatal complex traits, while germination parameters showed the lowest PI. We propose that soil moisture, salinity, and temperature are the most determinative and explanative variables of the variation between the three classified groups. We strongly believe that the phenotypic plasticity of *T*. *portulacastrum* will support species abundance and spread even under expected changes in climatic conditions, in contrast to the vulnerable traditional crops.

## 1. Introduction

Phenotypic plasticity is a key factor in the invasion process, promoting the adaptability and invasiveness of an alien species by increasing or maintaining population growth in unusual environmental conditions [1,2,3]. Plants’ functional traits should be examined to indicate which plasticity parameter(s) could play a role in the plant’s response to the global variation [4]. Investigations of trait variation along environmental gradients can also provide vital data for recognizing drivers of plant invasions and for deriving management strategies [5]. At the ecosystem level, the competition between wild species and invasive ones is considered a natural consequence, where the species with more remarkable adaptive plasticity may be more likely to survive in complicated, diverse habitats [6,7]. The plastic responses of the plant organs induced by the environment contribute to morphological and physiological behavioral changes among species inhabiting heterogeneous and variable conditions [7,8]. For certain morphological traits, phenotypic plasticity has been displayed to reflect genetic correlations. The traits that belong to the same set of characteristics are more genetically and phenotypically correlated than traits from different sets [9]. Monitoring the environmental effects on the micromorphology of the leaves and seeds indicates that the plants adapt their morphology to the new environmental conditions [10,11]. Many authors have reported the taxonomic significance of the macro- and micromorphological characteristics of Aizoaceae leaves and seeds [12,13,14,15,16,17,18].

The genus *Trianthema* L. (Aizoaceae, Sesuvioideae) comprises ~28 species classified into two subgenera: *Papularia*, and *Trianthema* [19]. Almost all species of the genus are C_4_ plants, which attain succulence with a high adaptive character under arid conditions [20,21]. *Trianthema portulacastrum* L. (*Syn. Trianthema monogynum* L.) is viewed as an aggressive, invasive weed. This species grows in several field crops, and is considered a major competitor for maize, cotton, mustard, soybeans, and some fruit trees. It was found to compete aggressively with soybeans, causing a reduction in the field productivity of 60%, and a reduction in the grain yield of 34% [22,23]. *Trianthema portulacastrum* is characterized by its facultative outcrossing, propagule pressure, high reproduction, huge seed bank, low dormancy, efficient seed dispersal, and thermotolerance [22]. All of these features support the spread, adaptability, and survival of this invasive weed in the face of climate change [24,25].

The European and Mediterranean Plant Protection Organization (EPPO) has added *T. portulacastrum* to the organization’s Alert List for 2020 due to the potential economic losses caused by this species. Additionally, CABI placed it in the “Invasive Species Compendium”, designating it as a threat to the crops and livelihoods worldwide [22]. On the other hand, *T. portulacastrum* has been reported for its benefits as a traditional medicine and fodder in various parts of the world [26].

In Egypt, Shaltout et al. [27] studied the distributional behavior and the growth performance of *T. portulacastrum* in the Nile Delta, indicating that its spread might lead to a severe ecological disaster. Recently, Ellmouni et al. [28] reported the significant variations between *T. portulacastrum* genotypes within the same population, along with the existence of sexual and clonal reproduction in the Fayoum Depression (FD), implying that local adaptation and phenotypic plasticity can help species invasion.

The Fayoum Depression is located in Egypt’s arid strip, with long, dry, hot summers and slightly warm winters [23]. It has a unique physical, environmental, and geographic location in the Western Desert of Egypt with a distinctive topography in terms of soil type, soil formation, drainage, irrigation, and agricultural land usage [29]. Additionally, it features a high evaporation rate, low seasonal rainfall with thermic soil temperature, and aridic soil moisture [30,31].

However, the effect of phenotypic plasticity on invasion success remains largely unknown, and it is vital to increase awareness about the upcoming invasive potential of *T. portulacastrum* [32,33]. Thus, this study aims to investigate the phenotypic plasticity of *T. portulacastrum* spreading in the Fayoum Depression by addressing its macro- and micromorphological and physiological traits, as well as to assess the phenotypic plasticity index of the species, which can be relevant for plant invasiveness.

## 2. Results

Descriptive data for the vegetative macromorphology, germination parameters, and photosynthetic pigment analysis are summarized in Table S1.

### 2.1. Vegetative Macromorphology

**Figure 1:**
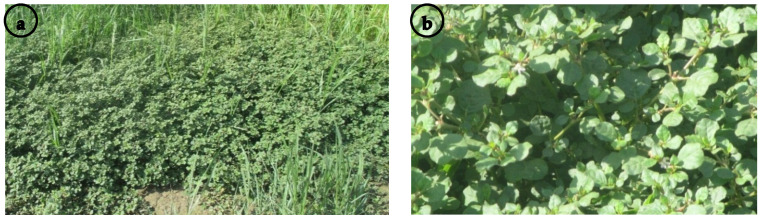
(**a**,**b**) Photographs of *Trianthema portulacastrum* in its natural habitat, taken by the authors.

*Trianthema portulacastrum* is a perennial succulent pubescent herb (Figure 1a). The stem is prostrate, with a number of secondary branches ranging from 3 to 17, while the number of tertiary branches of the stem varies from 3 to 9. The number of internodes at the primary branch is in the range of 5 to 15, while the number of internodes at the secondary branch varies from 3 to 12. The internode length of the primary branch ranges from 0.5 to 14.5 cm, whereas the internode length at the secondary branch varies from 0.3 to 10.5 cm.

The leaves are opposite and heterophyllous, with a membranous margin at the base. The single leaf petiole varies from 0.5 to 3 cm, the leaf size is 1.4−4.4 × 1.2−4.5 cm, and the area is in the range of 1.12 to 13.2 cm^2^. The single leaf shape is orbicular or ovate, with the apex being notched or rounded (Figure 1b). The leaf petiole varies from 0.2 to 1.3 cm, the leaf size is 0.9−2.3 × 0.5−1.8 cm, and the area is in the range of 0.3 to 2.76 cm^2^. The leaf shape is orbicular or ovate to elliptic, with the apex being apiculate to rounded.

### 2.2. Germination Analysis

The radical seedling length had a mean of 2.498 cm, where the minimum length (1.3 cm; sample 7) was observed in Fayoum, and the maximum length (4.43 cm; sample 24) was recorded in the northeast at Tamia. The average plumule length was 2.67 cm. The shortest and longest plumule lengths were recorded at Etsa in the south of the FD (1.73 cm—sample 3; and 3.53 cm—sample 4, respectively). The average length of the prophyllus was 0.37 cm, with the minimum value recorded in Etsa (0.26 cm; sample 2) and the maximum value (0.46 cm) found in Yousef El-Seddik (sample 30), Tamia (sample 25), and Fayoum (sample 8).

### 2.3. Photosynthetic Pigment Analysis

Descriptive statistics for the leaf pigment measurements showed that the average value of chlorophyll a (Chl a) was ~5 mg/g Fwt, with the minimum value being recorded in Ibshawy (2.61 mg/g Fwt; sample 27), while the maximum was observed in Senouris (8.06 mg/g Fwt; sample 20). The average value of chlorophyll b (Chl b) concentration was 1.58, with the lowest observed value in Ibshawy (0.68 mg/g Fwt; sample 28), while the highest value was exhibited in Fayoum (2.68 mg/g Fwt; sample 10). The average concentration of carotenoids was 1.14 mg/g Fwt, and the minimum and maximum were observed in Yousef El-Seddik (0.63 mg/g Fwt; sample 31) and Senouris (1.89 mg/g Fwt; sample 18), respectively.

The analyses reveal that the content of chlorophyll pigments differed significantly in the different habitats. This means that *T. portulacastrum* exhibited different plastic responses in different sites in FD.

Data of the three functional traits (vegetative morphology, germination analysis, and photosynthetic pigments analysis) for the 35 studied samples were accumulated to obtain a global perspective on changes in these functional traits.

**Figure 2:**
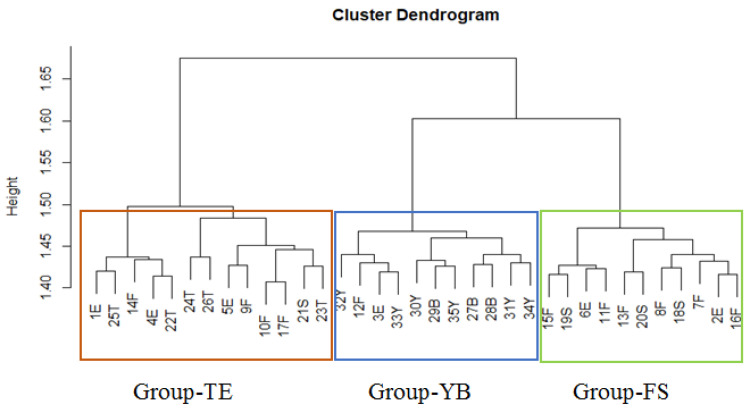
Agglomerative cluster analysis of 35 *Trianthema portulacastrum* samples subdivided into three groups (clusters) based on combined macromorphological characteristics, photosynthetic pigment analysis, and germination traits.

**Figure 3:**
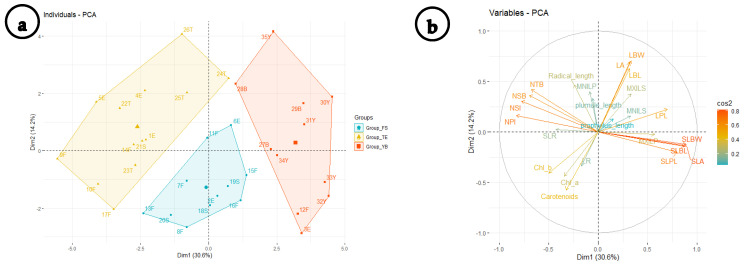
(**a**) Principal component analysis (PCA) of 35 *Trianthema portulacastrum* samples with 30.6% total variation explained along the first axis (Dim1) followed by a total 14.2% of variation explained along the second axis (Dim2). (**b**) Correlation between different variables and the first two components. Color intensity and length of the variable arrow in graph (**b**) is representing the importance of different variables where longer arrows with high color intensities are representing variables that contribute the most to the discrimination of the three groups.

The integration between the cluster analysis (Figure 2) and the principal component analysis (PCA) (Figure 3), along with the correlations of first and second dimensions for the most significant traits affecting the variation between groups and samples (Table 1), resulted in the discrimination of three main groups (clusters): Group-TE, Group-YB, and Group-FS. Group-TE comprised samples collected from Tamia, Etsa, Fayoum, and one sample from Senouris. Group-YB included samples from Ibshawy, Yousef El-Seddik, and one sample each from Etsa and Fayoum. Group-FS contained samples from the center of the FD (Fayoum and Senouris) and two from Etsa.

Comparing these traits for the three main groups using one-way ANOVA analysis showed significance for some vegetative macromorphological traits, as illustrated in Table 2. The following characteristics were significantly different between groups: number of secondary and tertiary branches, number of primary and secondary branch internodes, maximum internode length of the primary branch, minimum internode length of the secondary branch, single leaf blade length, width, L/W ratio and apex, and leaf petiole length. Furthermore, the comparison of *T. portulacastrum* radical length, chlorophyll pigment concentration (Chl a, Chl b, Chl a + Chl b), and the carotenoids for the three discriminated groups revealed significant differences (Table 2).

### 2.4. Micromorphological Analysis

Nine samples were selected from the total number of samples for the micromorphological leaf and seed analysis, in order to cover the variation within the three obtained groups (Group-TE, Group-YB, and Group-FS).

#### 2.4.1. Leaf Micromorphology

**Figure 4:**
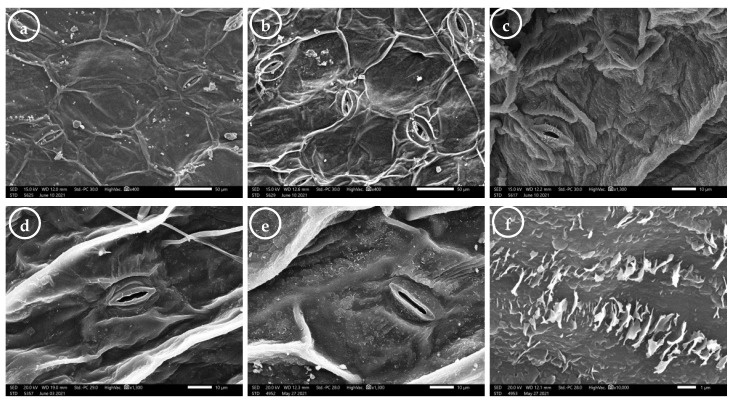
Scanning electron microscope (SEM) photomicrographs of *Trianthema portulacastrum* leaf surfaces: (**a**,**b**) general view showing the epidermal cell outline, anticlinal wall, curvature of the outer periclinal wall, and fine relief of the cell wall; (**c**–**e**) showing the fine relief of the cell wall, guard cell surface, pore shape, and epicuticular secretion type; (**f**) irregular thin platelets with an irregular margin. Abaxial leaf surface (**b**–**d**); Adaxial leaf surface (**a**,**e**,**f**). Scale bar= 50 µm (**a,b**); 10 µm (**c,d,e**); 1 µm (**f**).

The quantitative and qualitative leaf micromorphological characteristics were separately described for the abaxial and adaxial surfaces (Figure 4 and Supplementary Tables S2 and S3).

The epidermal cell outline was generally isodiametric or tetragonal to polygonal (Figure 4a–d). The epidermal cell size was 19.84−101.95 × 11.35−144.83 μm, and the area of these cells varied from 201.76 to 9940.53 µm^2^. The subsidiary cell size was in the range of 15.77−66.98 × 7.0−47.33 μm. The subsidiary cell area varied from 128.41 to 1569.26 µm^2^, with an average of 528.58 µm^2^. The anticlinal walls were irregularly curved in most samples (Figure 4c), while a straight AW was recorded for samples 12 and 28 (collected from Fayoum and Ibshawy, respectively) on both surfaces (Figure 4a). The relief of the cell wall was generally raised and smooth on both surfaces (Figure 4a–c). The curvature of the outer periclinal walls was flat (Figure 4b) or convex in the studied samples (Figure 4a,c). The fine relief of the cell wall was smooth (Figure 4a,b), slightly striate (Figure 4d,e), or striate—such as the leaves of sample 4 (from Etsa)—on both surfaces (Figure 4c). The epicuticular secretions were either film-like (in samples 2, 7, 28, and 33; from Etsa, Fayoum, Ibshawy, and Yousef El-Seddik, respectively; Figure 4d) or irregular thin platelets with an irregular margin of platelet height less than 1 μm (0.295−0.807) (in the other samples; Figure 4c,e,f).

**Figure 5:**
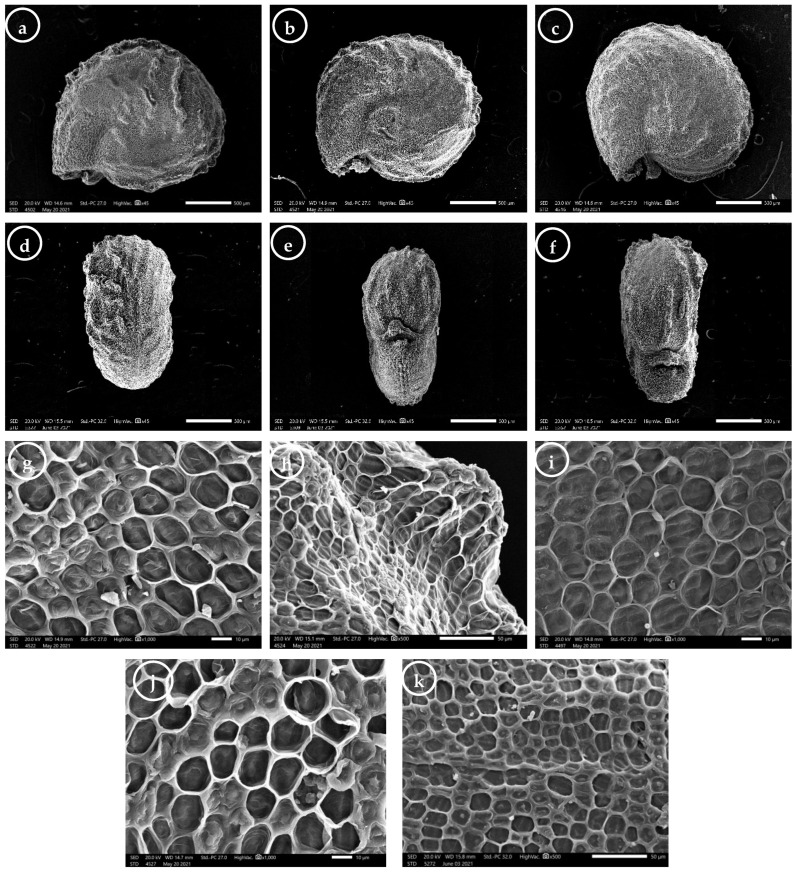
Scanning electron microscope (SEM) photomicrographs of *Trianthema portulacastrum* seeds: (**a**–**c**) general view of the lateral side of the seed, showing shape, apex, margin, and rib prominence; (**d**) general view of the dorsal side of the seed, showing number of ribs; (**e**,**f**) general view of the ventral side of the seed, showing the different hilum positions; (**g**–**k**) seed surface, showing the epidermal cell outline, anticlinal wall, relief of the cell boundary, curvature of the outer periclinal cell walls, shape of the periclinal walls’ protrusions, and fine relief of the cell wall. (**g**,**i**,**j**) Lateral side at the seed center; (**h**) lateral side at the seed edge; (**k**) dorsal side at the seed center. Scale bar = 500 µm (**a**–**f**); 50 µm (**h**,**k**); 10 µm (**g,i,j**).

Leaves were amphistomatic with raised tetracytic stomata (Figure 5a,b). The stomatal index (SI%) was higher on the AB surfaces than on the AD surfaces. Generally, the SI ranged from 16.67% (in sample 35, from Yousef El-Seddik) to 44.44% (in sample 4, from Etsa).

The guard cells’ surface was either smooth as in most samples (Figure 4d,e), or covered with very thin platelets (as in samples 4 and 25, from Etsa and Tamia, respectively) on both surfaces (Figure 4c). The opened stomatal complex size was 12.20−42.16 × 4.08−13.33 μm, whereas the stomatal pore size was 6.67−32.06 × 0.71−7.08 μm. The pore shape was an elliptic or linear slit on both surfaces (Figure 4d,e, respectively), although sample 25 from Tamia had both types on its AD surface. The closed stomatal complex size was 12.42−42.31 × 2.43−9.28 μm.

For the AB surfaces, the statistical analysis revealed that the stomatal complex length (opened state); stomatal pore length and L/W ratio; stomatal complex length, width, and L/W ratio (closed state), and the subsidiary cell length demonstrated significant differences between the three groups. For the AD surfaces, the stomatal complex length, width, and L/W ratio (opened state); stomatal pore length and width; stomatal complex length and width (closed state); epidermal cell length, width, and area; and the subsidiary cell area had significantly different values for the three groups (Table 3).

The statistical test (two-way ANOVA) revealed no significant variation between most of the measured characteristics of the AB and the AD surfaces for the three groups (Table S4). Nevertheless, the stomatal complex width (closed state) was significantly larger at the AB than the AD surfaces, while the stomatal complex L/W ratio (closed state) was higher at the AD than the AB surfaces for Group-TE. For Group-YB, the stomatal pore L/W ratio was significantly higher at the AB than the AD surfaces, and the stomatal complex width (closed state) was larger at the AD than the AB surfaces. In contrast, Group-FS displayed insignificant values for all of the studied characteristics.

#### 2.4.2. Seed Micromorphology

The quantitative and qualitative seed micromorphology traits are described for the central, rib position, dorsal and ventral side surfaces (Figure 5 and Tables S5–S7).

*Trianthema portulacastrum* seeds are exarillate, and their color is diverse, ranging from brownish-black to dull black within the seeds of the same individual. The seed size was 1.427−2.270 × 1.319−2.080 mm, and the seed L/W ratio varied from 0.88 to 1.36. The seed area ranged from 1.469 to 3.234 mm^2^, and the height was in the range of 0.655 to 0.936 μm. The shape was suborbicular (Figure 5a), reniform (Figure 5b), or rounded reniform (Figure 5c), and laterally compressed with a slightly convex lateral surface (Figure 5a–c). The seeds were transversely ribbed with 7–12 discontinuous (Figure 5d), moderate (Figure 5b,c) to highly prominent ribs (Figure 5a). The apex was rounded (Figure 5a–c), and the margin ranged from undulate (Figure 5a) to sinuate (Figure 5b,c). The hilum length varied from 0.107 mm to 0.343 mm. The hilum position was sub-basal (located at the second third) (Figure 5e) or basal (located at the last third of the seed) (Figure 5f).

The statistical analysis indicated that the seed length and area were significantly different between Group-TE (1.75 mm and 2.18 mm^2^, respectively) and Group-FS (1.87 mm and 2.50 mm^2^, respectively), while there were no significant differences between the former groups and Group-YB (1.85 mm and 2.35 mm^2^, respectively). The hilum length displayed a significant difference between Group-TE (0.178 mm) and Group-YB (0.225 mm), but Group-FS (0.196 mm) showed no significant difference.

The seed surface was described for the lateral (at the center and the edge positions; Table S6), dorsal, and ventral (at the center and at the base; Table S7) sides. The epidermal cell outlines were isodiametric tetra-, to octagonal (Figure 5g,j,k), oblong (Figure 5h), or rounded (Figure 5i), and arranged in rows. The isodiametric cells’ anticlinal walls were straight, with acute (Figure 5g) or curved angles (Figure 5j,k), while the oblong and rounded cells always had straight anticlinal walls (Figure 5h,i, respectively). The relief of the cell wall was moderately (Figure 5i) or highly raised (Figure 5g,h), and the curvature of the outer periclinal cell walls ranged from sunken flat to convex (Figure 5g–k). Some epidermal cells had protruded periclinal walls, and the shape of these protrusions was wedge-like (Figure 5g,i), dome-like (Figure 5h,j), or papillose (Figure 5k). The fine relief of the cell wall was slightly striate (Figure 5j,k) or striate (Figure 5g–i).

At the center of the lateral side, the epidermal cell count was in the range of 57 to 84 cells. The epidermal cell size was 10.15−27.02 × 7.20−19.17 μm, and its area varied from 68.97 to 337.57 μm^2^. At the edge, the cell size was much larger than that of the cells mentioned above, at 17.12−43.27 × 10.27−24.29 μm, with the area ranging from 158.88 to 741.45 μm^2^.

The one-way ANOVA for the seeds’ lateral sides showed significant variation in the epidermal cell count at the center and the epidermal cell width at the edge (Table 3).

For the dorsal side shape of the periclinal walls, protrusions were dome-like, and the fine relief of the cell wall was slightly striate. The epidermal cell count ranged from 41 to 61 cells (in samples 2 and 33, respectively; from Etsa and Yousef El-Seddik, respectively), and the epidermal cell size was 9.82−28.85 × 7.97−18.49 μm, with an area ranging from 65.65 to 385.90 μm. At the center, the epidermal cell count varied from 26 to 59 cells (in samples 21 and 4, respectively; from Senouris and Etsa, respectively); the epidermal cell size was 9.60−26.19 × 6.31−20.19 μm, and its area varied from 54.02 to 292.66 μm.

The two-way ANOVA of the epidermal cell measurements (length, L/W ratio, and area) at the edge of the seeds exhibited a significant difference, with much larger cells than those located at the other positions on the seed surface. Meanwhile, the epidermal cell parameters were insignificant for the three studied groups between the central, dorsal, and ventral seed positions (Table S8).

### 2.5. Phenotypic Plasticity Index (PI)

**Figure 6:**
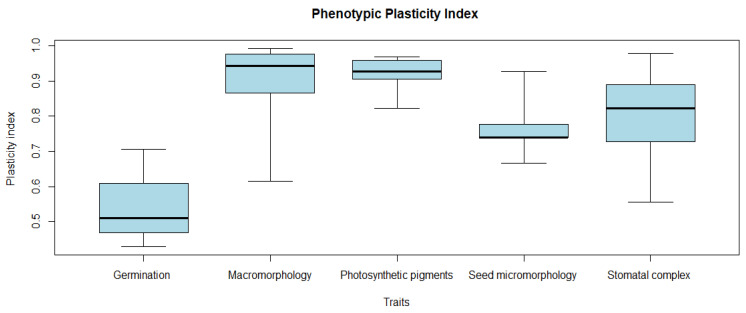
Boxplots showing phenotypic plasticity index (PI) distribution for each of the measured traits along samples of *Trianthema portulacastrum*.

Phenotypic plasticity index values for all of the investigated germination, macromorphology, photosynthetic pigments, seed micromorphology, and stomatal complex traits are shown in Table 4. Boxplots with the comparisons between the studied samples showed a greater mean PI for the photosynthetic pigments and macromorphological characteristics (0.91 and 0.90, respectively), followed by the values obtained from micromorphological characteristics (stomatal complex traits, 0.80; seed micromorphology, 0.77); in contrast, germination data showed the lowest PI (0.54) (Figure 6).

**Figure 7:**
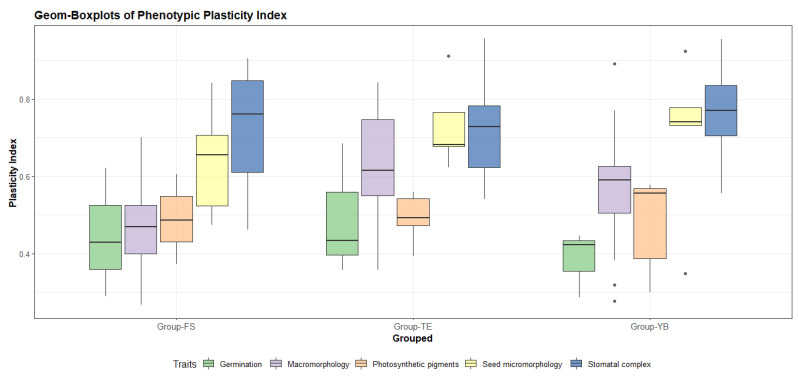
Boxplots showing phenotypic plasticity index (PI) distribution for each of the measured traits among the three identified groups.

The plasticity index showed variable values between the three analyzed groups (Table 5). Boxplots comparing these groups exhibited the highest PI for micromorphological traits, seed micromorphology and stomatal complex for the three groups, with values of 0.77 and 0.70, respectively, for Group-YB; 0.72 and 0.64, respectively, for Group-FS; and 0.71 and 0.73, respectively, for Group-TE. Meanwhile, macromorphological data and photosynthetic pigment data showed moderate PI for the three groups, with values of 0.62 and 0.49, respectively, for Group-TE; 0.56 and 0.48, respectively, for Group-YB; and 0.45 and 0.48, respectively, for Group-FS. The germination data showed the lowest PI (0.49, 0.44, and 0.38 for Group-TE, Group-FS, and Group-YB, respectively). The final results confirmed that *T. portulacastrum* had broad variations in phenotypic plasticity (Figure 7). 

## 3. Discussion

The Fayoum Depression has an elevation range from −52 m below sea level to 141 m above sea level; its highest point lies at the southeastern part of the depression, where it meets the Nile, while the lowest point lies at the northwestern part of the depression, near Qarun Lake. In general, elevation increases gradually from south to north. The salinity level is also related to the elevation, where salinity ranges from below 2 ds/m at the highest elevation point to more than 16 ds/m at Qarun Lake and its vicinity—which is unsurprising, as Qarun Lake has received the agricultural wastewater of the whole depression for many decades [34].

Invasive species can sustain normal development by using various morphological and physiological plasticity mechanisms, allowing them to adapt to diverse environmental conditions [35]. The present study aimed to assess the phenotypic plasticity of *Trianthema portulacastrum* in terms of germination, macromorphology, photosynthetic pigments, seed micromorphology, and stomatal complex traits. Our results showed that the 35 samples collected from different locations in the Fayoum Depression could be classified into three main groups, each with distinctive characteristics that distinguish them from the rest.

Group-FS had the highest photosynthetic pigment contents—including Chl a, Chl b, and carotenoids—in comparison to the other two groups. Chl b accumulation facilitates light absorption, capturing different wavelengths than those captured by Chl a and transferring them to the reaction centers [36]. This may contribute to the acclimatization of *T. portulacastrum* to saline regions, since Chl b is the main component of the LHC protein, which acts as a complex antenna in the transfer of energy to the reaction center photosystem II [36]. On the macromorphological level, significant traits results showed moderate values compared to the other two groups. Leaf area and radical length also showed moderate levels. Out of the 36 measured parameters in the stomatal complex, 18 were significant. In general, this group had the highest average values in most leaf measurements, but the lowest in the seed micromorphological traits. Moreover, the epicuticular secretions were film-like. This group mainly represents samples collected from Fayoum and Senouris. Environmentally, this group is characterized by high clay content (>50%), moderate organic matter (1.6%), moderate elevation (15–30 m above sea level), low-to-moderate salinity levels (2–6 ds/m), and moderate calcium carbonate content (3–5%) [37,38]. This group represents a fertile habitat with high clay content, low water and soil salinity, good water availability, and high elevation.

Group-TE had the largest number of secondary and tertiary branches and internodes, while on the other hand, it had the smallest leaf area and petiole length, and longer radical length in relation to the other groups. This may be attributed to the leaf eco-physiological traits, which tend to reduce leaf area and radical length in order to conserve water during stressed conditions, because water might be available at deeper soil layers. In response to salt stress, leaf characteristics were linked with immediate physiological processes and pigments enhancing plastic response [39]. Moderate Chl a, Chl b, and carotenoid contents were observed in this group compared to the other two groups. Leaf and seed micromorphology measurements showed moderate values in this group comparing to the other two groups. The micromorphological leaf traits of *T. portulacastrum* in this group were highly correlated with the environment, supporting the observations of Chen et al. [40]. The epicuticular waxes are essential structural elements of the plant surface, reflecting the interaction between plants and their environment [41]; they are considered a multifunctional surface to limit uncontrolled water loss, protect against microbes and insects, and increase plants’ drought and salinity stress response [42]. The relatively higher density of leaf waxes and the presence of thin irregular platelets on the guard cells for the members of this group can be attributed to salinity and drought stresses [38]. The salinity reduced the epidermal cell size parameters of the studied samples—such as length, width, and area—similar to the results of El-monim [43] and Zörb et al. [44]. This group represents samples from Tamia, Etsa, and Fayoum, and it is characterized by high sand content (>50%), moderate organic matter (1.5%), high calcium carbonate content (13–20%), moderate-to-highly salinity (5–10 ds/m), and a moderate-to-low elevation level—from −15 m below sea level to 15 m above sea level [37,38]. This group represents a mid-level habitat with moderate water and soil salinity, low water availability, high sand content, and moderate elevation.

Group-YB had the lowest number of secondary and tertiary branches and internodes, while on the other hand, it has the largest leaf area, longest petiole length, and shorter radical length compared to the other groups, due to the high-salinity water and high evaporation rate [45,46]. The unexpected larger leaf area in this group could reflect the ability of these samples to maintain a sizeable transpiring surface area, which could be part of an integrated mechanism of the whole-plant acclimation to salt stress [47,48]. Photosynthetic pigment contents showed that this group had the lowest content of Chl a, Chl b, and carotenoids. The inhibition of chlorophyll synthesis is a typical symptom of oxidative stress, along with the activation of its degradation by the enzyme chlorophyllase [49]. Reduction in chlorophyll concentrations indicated a photoprotection mechanism that reduced light absorption by lowering chlorophyll contents [50]. Our results revealed significant decreases in carotenoids in salty habitats, which is consistent with the previous findings of Ghanem et al. [51]. Carotenoids accumulate solar energy for the photosynthetic process, and they also quench chlorophyll triplet states [52]. The decrease in carotenoid contents indicated that the protection provided by carotenoids was not one of the most relevant mechanisms under salt stress [53]. Leaf and seed micromorphology results showed that this group had the lowest measurements compared to the TE and FS groups. Moreover, the lowest stomatal index on both leaf surfaces was noticed. Bertolino et al. [54] suggested that reduction in the number of stomata might be ascribed to drought stress. This group represents samples collected from Yousef El-Seddik and Ibshawy, and is characterized by low elevation levels (−50 m below sea level to 0 m), moderate-to-extremely saline conditions (6–32 ds/m), moderate-to-high organic matter (1.3 to 2.2%), and high calcium carbonate content (11–18%) [34,37]. This group represents a severe habitat, with high water and soil salinity, low water availability, and low elevation.

The seed morphology and its surface microstructure are important tools for elucidating the impact of various environmental factors on the phenotypic variability of some invasive species occupying a wide range of habitats [55]. It was remarkable that *T. portulacastrum* seed color, size, and shape were diversified within the seeds of the same individual. Singh [56] noted that Aizoaceae show a specific epidermal cell arrangement and orientation of smaller and larger cells, known as a centrospermoid pattern. Accordingly, the seed edge showed the highest average values for all characteristics (epidermal cell length, length/width ratio, and area) in comparison to the lateral (center), dorsal, and ventral sides within all groups, while there was no variation among seed edges between any of the groups. 

A better understanding of introduced species’ invasive ability and its future invasive potential can be achieved by investigating their phenotypic plasticity [57]. Plastic patterns of biomass distribution may help plant species to invade more successfully by searching for resources more efficiently as environmental conditions change [58]. The plasticity index of morphological and photosynthetic pigment traits showed the highest variation between the three groups (>90%) but the lowest variation within each of three identified groups (45–60%). On the other hand, stomatal complex and seed micromorphology plasticity indices were constant between groups and within groups, at around 65–80%. Germination data remained low between groups and within groups, at around 40–55%. The highest PI values for the various traits explain the higher responsiveness of *T. portulacastrum* to different environments, such as its ability to spread and grow under variable habitats in the FD [59]. In sum, its seeds have a greater reproductive capacity and dispersion potency, and can endure high soil temperatures [22]. Furthermore, various studies have revealed evidence of the impact of soil moisture, salinity, and drought stress on trait variations to facilitate plant invasion and adaptation to diverse habitats [35,60]. Invasion success may be influenced by phenotypic plasticity and the evolution of reproductive characteristics, which are related to the seeds’ polymorphism and their dispersal behavior [61]. Accordingly, these features are required for invasive plants because they are related to dispersal tactics and systems to cope with environmental stress, maximizing their performance and establishment in the new environment [55,62]. This could also be related to the susceptibility of seeds to the various abiotic and biotic conditions [63]. These variables are considered the most significant for the three groups in the study area, and ameliorate the plasticity of *T. portulacastrum* in the FD. In agreement with this, Bufford et al. [39] revealed the positive correlation of multi-trait plasticity to drought with the possible adaptation of invasive alien species.

In conclusion, the present study shows that *Trianthema portulacastrum* has a high plasticity index, which may allow the invasive species to adapt, flourish, and spread in different habitats—even under drought and saline conditions. The three identified groups in the present study showed wide variation in the different traits and environmental conditions associated with each group, which may explain the widespread distribution of the invasive species not only at the level of Fayoum, but throughout the whole country of Egypt, as well as neighboring countries.

Further research to study the ability of *T. portulacastrum* to adapt and spread under expected changes in climatic conditions would help in devising future scenarios to hinder the invasiveness of this species against traditional crops. In light of the reported high phenotypic plasticity, it would be a win–win situation to use this species and get rid of it at the same time.

## 4. Materials and Methods

### 4.1. Study Area

The Fayoum Oasis is a natural topographic depression, and it is regarded as one of the most essential Egyptian productive agronomic areas since the Eocene limestone plateau. The FD is located in the west of the Nile Valley, nearly 90 km southwest of Cairo, occupying the northern part of the Western Desert [29]. The Fayoum Depression is a unique depression that lies on the fringes of the Libyan Desert—one of the most arid deserts in the world—and lies over the largest aquifer in the world: the Nubian Sandstone Aquifer System. At the same time, it is connected to the Nile Valley via a small canal named “Bahr Youssef” [34,64]. All of these features are combined to build widely different habitats based on water availability, water and soil salinity, and elevation [65].

### 4.2. Plant Materials

**Figure 8:**
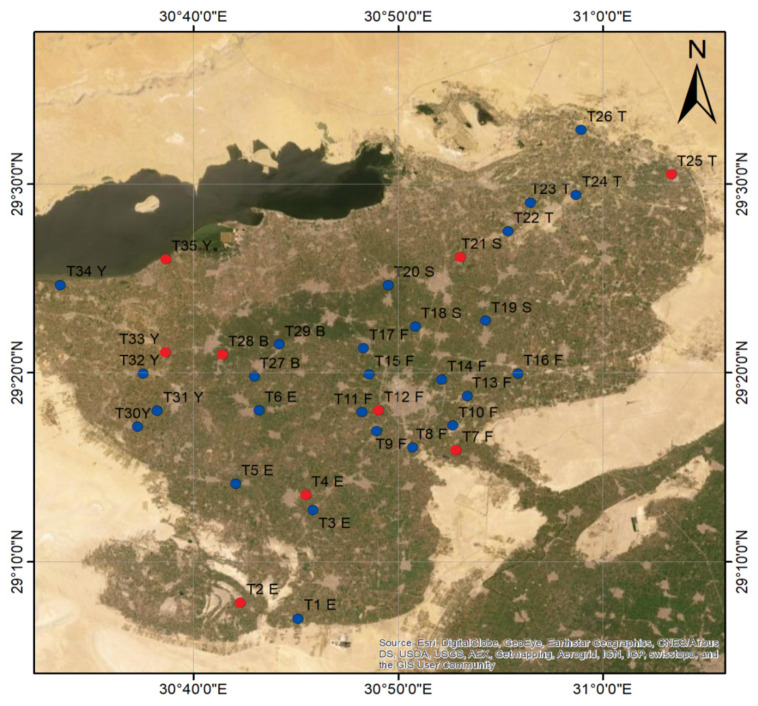
Geographical distribution map of 35 *T. portulacastrum* samples collected from the Fayoum Depression, Egypt. The red dots indicate the nine samples selected for leaf and seed micromorphological (SEM) studies, based on the cluster analysis of macromorphological characteristics.

Thirty-five samples of *T. portulacastrum* were collected from six districts: Etsa, Fayoum, Senouris, Tamia, Ibshawy, and Yousef El-Seddik. The collected samples were randomly distributed in the six districts (Figure 8). They were collected from all habitats (mesophytes, halophytes, and xerophytes), field crops, roadsides, and wastelands. *Trianthema portulacastrum* also exists at varying elevations in the FD, ranging from −47 to 27 m. Collection sites and sampling information of the thirty-five samples are represented in Table S9. Voucher specimens were deposited in Fayoum University Herbarium, Fayoum, Egypt. 

### 4.3. Morphometric, Germination, and Photosynthetic Pigment Analysis

Twenty-two stem and leaf morphological characteristics were investigated for 35 herbarium samples of *T. portulacastrum*. As described by Ash [66] the leaf area was calculated using the following equation (leaf length × leaf width × 2/3). The quantitative treatments were measured using ImageJ (1.51j8), and the terminology of Shaltout et al. [27] was used.

*Trianthema portulacastrum* seeds collected from the six districts were germinated for 14 days to determine the variations in growth parameters. The chlorophyll pigments were determined by the Lichtenthaler–Buschmann method [67].

### 4.4. Leaf and Seed Micromorphology (SEM)

The surface micromorphology of leaves and seeds was investigated for nine *T. portulacastrum* samples (2, 4, 7, 12, 21, 25, 28, 33, and 35), as illustrated with red dots in Figure 8.

These samples were selected from the three clusters that resulted from the multivariate analysis of the macromorphological characteristics. The samples were fixed directly onto stubs with double-sided adhesive tape, and then were coated for 5 min with gold in a Polaron JFC-1100E coating unit. Leaves and seeds were examined and photographed with a JEOL JSM-IT200 (Japan) scanning electron microscope (Faculty of Science, Alexandria University, Alexandria, Egypt).

For the leaf micromorphology, a total of fifty quantitative and qualitative characteristics were studied for both abaxial (AB) and adaxial (AD) leaf surfaces. The quantitative traits included stomatal length and width (in the closed and opened states); stomatal pore length and width; epidermal cell length, width, and area; and the subsidiary cell length, width, and area. The stomatal index (SI%) was calculated using the following formula:

$$Stomatal\;Index\;\left(\%\right)=\frac{\mathrm{Number}\;\mathrm{of}\;\mathrm{stomata}\;\mathrm{per}\;\mathrm{unit}\;\mathrm{area}}{\mathrm{Number}\;\mathrm{of}\;\mathrm{stomata}\;\mathrm{per}\;\mathrm{unit}\;\mathrm{area}+\mathrm{Number}\;\mathrm{of}\;\mathrm{epidermal}\;\mathrm{cells}\;\mathrm{per}\;\mathrm{unit}\;\mathrm{area}}\times 100$$

The terminology of Barthlott et al. [41] and Singh [56] was used.

For the seed micromorphology, forty-three characteristics were studied for the dorsal, lateral, and ventral seed sides. The studied samples were evaluated for diagnostic seed surface features such as hilum position, epidermal cell outline and size, anticlinal wall, and outer periclinal wall, using the terminology of Hassan et al. [15] and Zeng et al. [68]. The quantitative leaf and seed measurements were taken using ImageJ (1.51j8) [69].

### 4.5. Plasticity Index (PI)

The phenotypic plasticity index (PI) was calculated separately for the measured plant traits—germination data, macromorphology, photosynthetic pigments, stomatal complex traits, and seed micromorphology [60]. The difference between the minimum and maximum values was divided by the maximum value to produce the index [70]. Higher PI values, which are closer to one, imply that the variable is more plastic [70]. The mean plasticity index (PI) was derived by averaging the plasticity index for all leaf characteristics studied. The PI has the benefit of comparing variables with various units and contrasting ranges [32,71].

### 4.6. Statistical Analysis 

Data were analyzed using various tools, including Excel 365, Minitab ver. 20, and R [72]. Data were cleaned before running any statistical analyses. Missing data and mistyping errors were checked. Descriptive statistics, including the mean and standard deviation, were calculated for each factor. Inferential multivariate statistics were used to group the 35 localities into a number of homogeneous groups based on different measured traits. Agglomerative cluster analysis using Euclidean distance measurement and Ward’s linkage method was carried out after standardization and scaling of various variables using R [73]. Principal component analysis (PCA)—an ordination technique—was used to find the correlation between different clusters and measured variables along the first two axes of the PCA. PCA was carried out after implementation of the two packages “Factoextra” and “FactoMineR “ in R for visualizing the joint biplot and ordination results [73].

Inferential univariate statistics were used to compare the results of different groups (clusters obtained from the multivariate analyses). Germination, macromorphological, and photosynthetic pigment data were analyzed and compared between the resultant groups. All variables’ parametric assumptions were tested. Box–Cox transformation was applied for non-normal dependent variables via the optimal *λ* method using Minitab 19. Different comparisons were conducted using one-way and two-way analysis of variance (ANOVA) under general linear model. Results showed a good fit for different models, while normal residual probability plots showed a linear attitude for all analyses after data transformation. *p*-values were considered significant at α < 0.05. Post hoc analyses of the interactions between all groups were conducted using Tukey’s test for pairwise comparisons. Results of the post hoc analyses are represented as letters, where groups that share the same letters are not significantly different, while different letters express significant differences between different groups. Data representation—including boxplots, PCA, and cluster dendrograms—was carried out using R and Excel 365. Similar procedures were applied to compare the results of the seed morphology and stomatal complex traits after selecting representative samples from each cluster obtained in the multivariate analyses.

## Figures and Tables

**Table 1:** Pearson correlation (r) for the significantly correlated variables (with *p*-value of <0.001) with the first two components of PCA. Variables are sorted in a descending order according to correlation value.

PCA Dimension 1		PCA Dimension 2	
Traits	r	Traits	r
Single leaf blade width	0.89	Leaf blade width	0.71
Single leaf area	0.89	Leaf area	0.69
Single leaf blade length	0.88	Leaf blade length	0.63
Single leaf petiole length	0.80	Radical length	0.47
Leaf petiole length	0.7	Number of tertiary branches	0.42
Maximum internode length of primary branch	0.57	Maximum internode length of primary branch	0.4
Leaf blade width	0.33	Maximum internode length of secondary branch	0.38
Chl a	−0.34	Number of secondary branches	0.36
Single leaf L/W ratio	−0.43	Leaf area	−0.34
Chl b	−0.5	Chl b	−0.41
Number of tertiary branches	−0.68	Chl a	−0.44
Number of secondary branches	−0.7	Carotenoids	−0.58
Number of secondary branch internodes	−0.78		
Number of primary branch internodes	−0.83		

**Table 2:** One-way ANOVA results and pairwise comparisons among the three groups of *Trianthema* *portulacastrum*, showing the significant vegetative macromorphology, germination, and photosynthetic pigment traits. Groups that share same letters are non-significant, while different letters represent significantly different groups.

Traits		Group-TE	Group-YB	Group-FS
Vegetative macromorphology	Number of secondary branches	11.231 ± 3.004 ^a^	5.909 ± 1.758 ^b^	7.909 ± 2.343 ^b^
	Number of tertiary branches	7 ± 1.871 ^a^	4.364 ± 1.567 ^b^	4.455 ± 1.036 ^b^
	Number of primary branch internodes	12.615 ± 2.293 ^a^	7.091 ± 1.758 ^c^	9.727 ± 2.37 ^b^
	Number of secondary branch internodes	8.692 ± 2.213 ^a^	5.273 ± 1.954 ^b^	6.273 ± 1.618 ^b^
	Maximum internode length of primary branch (cm)	7.077 ± 1.813 ^b^	9.818 ± 2.205 ^a^	8.591 ± 1.855 ^ab^
	Minimum internode length of primary branch (cm)	0.854 ± 0.382 ^ab^	0.973 ± 0.338 ^a^	0.6273 ± 0.2195 ^b^
	Single leaf blade length (cm)	2.308 ± 0.522 ^c^	3.673 ± 0.494 ^a^	3.1455 ± 0.3297 ^b^
	Single leaf blade width (cm)	1.885 ± 0.483 ^c^	3.491 ± 0.632 ^a^	2.7182 ± 0.2994 ^b^
	Single leaf L/W ratio	1.247 ± 0.1994 ^a^	1.0655 ± 0.1204 ^b^	1.1652 ± 0.1352 ^ab^
	Single leaf area (cm^2^)	3.018 ± 1.363 ^c^	8.695 ± 2.516 ^a^	5.725 ± 1.014 ^b^
	Leaf petiole length (cm)	0.4077 ± 0.2178 ^b^	0.79 ± 0.2767 ^a^	0.4818 ± 0.1079 ^b^
Germination	Radical length (cm)	2.97 ± 0.866 ^a^	2.139 ± 0.459 ^b^	2.3 ± 0.735 ^ab^
Photosynthetic pigments	Chl a (mg/g Fwt)	5.123 ± 1.135 ^ab^	4.097 ± 1.107 ^b^	5.772 ± 1.4 ^a^
	Chl b (mg/g Fwt)	1.73 ± 0.482 ^a^	1.1509 ± 0.305 ^b^	1.846 ± 0.459 ^a^
	Chl a + Chl b (mg/g Fwt)	6.853 ± 1.479 ^a^	5.248 ± 1.379 ^b^	7.618 ± 1.807 ^a^
	Carotenoids (mg/g Fwt)	1.1369 ± 0.2794 ^ab^	0.9552 ± 0.289 ^b^	1.346 ± 0.356 ^a^
	Chl a/b (mg/g Fwt)	3.046 ± 0.538 ^b^	3.592 ± 0.451 ^a^	3.158 ± 0.412 ^ab^

**Table 3:** One-way ANOVA results and pairwise comparisons among the three groups of *Trianthema* *portulacastrum*, showing the significant leaf and seed micromorphological traits. Groups that share same letters are non-significant while different letters represent significantly different groups.

Traits			Group-TE	Group-YB	Group-FS
Leaf micromorphology	Abaxial surface	Stomatal complex length (opened) (µm)	20.039 ± 1.782 ^ab^	17.908 ± 2.662 ^b^	24.75 ± 9.25 ^a^
		Stomatal pore length (µm)	11.418 ± 2.303 ^ab^	10.587 ± 2.591 ^b^	16.36 ± 8.44 ^a^
		Stomatal pore L/W ratio	8.8 ± 4.18 ^a^	5.182 ± 2.355 ^b^	7.726 ± 3.216 ^a^
		Stomatal complex length (closed) (µm)	16.573 ± 1.716 ^ab^	15.066 ± 1.547 ^b^	18.98 ± 5.63 ^a^
		Stomatal complex width (closed) (µm)	5.123 ± 0.714 ^a^	4.096 ± 0.535 ^b^	4.59 ± 1.515 ^b^
		Stomatal complex L/W ratio (closed)	3.288 ± 0.533 ^b^	3.742 ± 0.65 ^ab^	4.271 ± 0.864 ^a^
		Subsidiary cell length (µm)	34.78 ± 6.01 ^a^	27.96 ± 8.19 ^ab^	29.81 ± 12.41 ^a^
	Adaxial surface	Stomatal complex length (opened) (µm)	21.98 ± 5.2 ^a^	17.262 ± 2.814 ^b^	23.82 ± 6.27 ^a^
		Stomatal complex width (opened) (µm)	6.363 ± 3.487 ^b^	5.396 ± 0.676 ^b^	8.247 ± 2.087 ^a^
		Stomatal complex L/W ratio (opened)	3.844 ± 1.207 ^a^	3.211 ± 0.457 ^ab^	2.945 ± 0.593 ^b^
		Stomatal pore length (µm)	13.72 ± 4.24 ^a^	9.267 ± 2.345 ^b^	16.46 ± 5.79 ^a^
		Stomatal pore width (µm)	1.802 ± 0.714 ^b^	1.764 ± 0.63 ^b^	3.125 ± 1.607 ^a^
		Stomatal complex length (closed) (µm)	17.433 ± 3.372 ^b^	17.32 ± 3.44 ^ab^	22.76 ± 7.92 ^a^
		Stomatal complex width (closed) (µm)	4.296 ± 1.017 ^b^	4.529 ± 1.282 ^b^	6.025 ± 0.852 ^a^
		Epidermal cell length (µm)	37.64 ± 16.39 ^b^	31.46 ± 3.72 ^b^	58.48 ± 20.37 ^a^
		Epidermal cell width (µm)	32.8 ± 22.13 ^b^	23.84 ± 7.34 ^b^	56.72 ± 28.24 ^a^
		Epidermal cell area (µm^2^)	1435 ± 1553 ^b^	685 ± 259 ^b^	2992 ± 2119 ^a^
		Subsidiary cell area (µm^2^)	677.9 ± 415 ^a^	352.1 ± 132.2 ^b^	637.8 ± 405.1 ^a^
Seed micromorphology	Lateral side (center)	Epidermal cell count	65.33 ± 3.21 ^ab^	74 ± 5.66 ^a^	61.25 ± 3.77 ^b^
	Lateral side (edge)	Epidermal cell width (µm)	15.073 ± 2.763 ^ab^	13.607 ± 2.548 ^b^	16.436 ± 3.372 ^a^

**Table 4:** Descriptive plasticity index (PI) values for all of the studied data of *Trianthema portulacastrum*, showing significant comparisons between them. Groups that share same letters are non-significant while different letters represent significantly different groups.

Traits		Mean ± SD	SE Mean	Minimum	Q1	Median	Q3	Maximum	IQR
Plasticity index	Germination data	0.5483 ± 0.1431 ^c^	0.083	0.428	0.428	0.509	0.707	0.707	0.278
	Macromorphological data	0.9051 ± 0.0993 ^a^	0.023	0.615	0.861	0.943	0.978	0.994	0.116
	Photosynthetic pigment data	0.9185 ± 0.0525 ^ab^	0.021	0.822	0.884	0.927	0.962	0.969	0.077
	Seed micromorphology	0.7703 ± 0.6667 ^bc^	0.043	0.667	0.703	0.740	0.853	0.927	0.149
	Stomatal complex traits	0.8079 ± 0.5555 ^b^	0.025	0.556	0.727	0.823	0.898	0.979	0.171

**Table 5:** Descriptive statistics of the plasticity index (PI) for the studied traits, showing pairwise comparisons among the three groups of *Trianthema portulacastrum*. Groups that share same letters are non-significant while different letters represent significantly different groups.

Groups	Traits	Mean ± SD	SE Mean	Min	Q1	Median	Q3	Max	IQR
Group-TE	Germination data	0.4918 ± 0.171 ^b^	0.0987	0.3572	0.3572	0.4339	0.6842	0.6842	0.327
	Macromorphological data	0.6214 ± 0.1479 ^abc^	0.0348	0.3571	0.5458	0.6152	0.7795	0.8421	0.2337
	Photosynthetic pigment data	0.4937 ± 0.0616 ^c^	0.0251	0.3933	0.4508	0.4923	0.556	0.5597	0.1053
	Seed micromorphology	0.7317 ± 0.1127 ^ab^	0.0504	0.6232	0.6501	0.681	0.8386	0.9114	0.1885
	Stomatal complex traits	0.7184 ± 0.1169 ^a^	0.0275	0.5411	0.6148	0.7273	0.79	0.9556	0.1751
Group-YB	Germination data	0.3847 ± 0.0864 ^c^	0.0499	0.2858	0.2858	0.4223	0.446	0.446	0.1601
	Macromorphological data	0.5687 ± 0.1531 ^bc^	0.0361	0.2762	0.4861	0.5901	0.6354	0.8913	0.1493
	Photosynthetic pigment data	0.4821 ± 0.1293 ^bc^	0.0528	0.2996	0.3243	0.5553	0.5735	0.5776	0.2492
	Seed micromorphology	0.7044 ± 0.2135 ^a^	0.0955	0.3485	0.5399	0.7402	0.851	0.9239	0.3111
	Stomatal complex traits	0.7783 ± 0.1042 ^ab^	0.0246	0.5555	0.6966	0.7696	0.8549	0.9545	0.1583
Group-FS	Germination data	0.4462 ± 0.167 ^b^	0.0964	0.2887	0.2887	0.4285	0.6213	0.6213	0.3327
	Macromorphological data	0.4586 ± 0.1196 ^b^	0.0282	0.2666	0.4	0.4694	0.5429	0.7	0.1429
	Photosynthetic pigment data	0.4885 ± 0.0886 ^b^	0.0362	0.3732	0.4022	0.486	0.5773	0.6061	0.175
	Seed micromorphology	0.6402 ± 0.1468 ^a^	0.0657	0.4744	0.4991	0.6547	0.774	0.8418	0.2749
	Stomatal complex traits	0.721 ± 0.1503 ^ab^	0.0354	0.4606	0.5875	0.7604	0.852	0.9047	0.2645

## Data Availability

The data presented in this study are available in this article and the Supplementary Materials.

## Supplementary Materials

The following are available online at https://www.mdpi.com/article/10.3390/plants11010077/s1: Table S1: Descriptive statistics of vegetative macromorphology, germination, and photosynthetic pigment traits of *Trianthema portulacastrum*, Table S2: Quantitative characteristics of abaxial and adaxial leaf micromorphology of *Trianthema portulacastrum*, Table S3: Qualitative characteristics of abaxial and adaxial leaf micromorphology of *Trianthema portulacastrum*, Table S4: Two-way ANOVA of abaxial and adaxial leaf micromorphological characteristics of *Trianthema portulacastrum*, Table S5: Descriptive statistics of *Trianthema portulacastrum* seed micromorphology, Table S6: Qualitative characteristics of *Trianthema portulacastrum* seed micromorphology for the lateral side, Table S7: Qualitative characteristics of *Trianthema portulacastrum* seed micromorphology for the dorsal and ventral sides, Table S8: Two-way ANOVA of *Trianthema portulacastrum* seed micromorphology, Table S9: Collection sites and sampling information of thirty-five samples representing *Trianthema portulacastrum* collected from Fayoum Depression (FD), Egypt.
