# Supplementary material for: Investigating the Phenotypic Plasticity of the Invasive Weed Trianthema portulacastrum L
Source: Plants (Basel). 2021 Dec 27;11(1):77. doi: 10.3390/plants11010077 (PMC8747563; doi:10.3390/plants11010077)
Supplement: Supplementary file 1 [file plants-11-00077-s001.zip › plants-1486414-supplementary.pdf]

plants

MDPI

Investigating the Phenotypic Plasticity of an Invasive Alien Weed *Trianthema portulacastrum* L.

Marwa A. Fakhr[1,2], Yasser S.A. Mazrou[3,4], Faten Y. Ellmouni[1]*, Al-Baraa El-Saied[5], Mohamed Elhady[6], Amr El Kelish[7], Iman H. Nour[8]*

[1] Botany Department, Faculty of Science, Fayoum University, 63514, Fayoum, Egypt; maa29@fayoum.edu.eg (M.A.F.); fyl00@fayoum.edu.eg (F.Y.E.).

[2] Plant Protection and Biomolecular Diagnosis Department, Arid Lands Cultivation Research Institute, City of Scientific Research and Technological Applications (SRTA-City), New Borg El-Arab City, Alexandria, 21934, Egypt.

[3]Business Administration Department, Community College, King Khalid University, Guraiger, Abha 62529, 10 Saudi Arabia; ymazrou@kku.edu.sa (Y.M)

[4]Faculty of Agriculture, Tanta University, Tanta 31512, Egypt

[5] Botany and Microbiology Department, Faculty of Science, Al-Azhar University, Cairo, Egypt; albraa.mahmoud@azhar.edu.eg; (A.B.E.).

[6] Botany and Microbiology Department, Faculty of Science, Al-Azhar University, Cairo, Egypt; Elhadymohamed566@Yahoo.com

[7] Botany Department, Faculty of Science, Suez Canal University, Ismailia 41522, Egypt; amr.elkelish@science.suez.edu.eg,(A.E.).

[8] Botany and Microbiology Department, Faculty of Science, Alexandria University, 21511 Alexandria, Egypt; Iman.nour@alexu.edu.eg (I.H.N.).

* Corresponding author: fyl00@fayoum.edu.eg (F.Y.E), Iman.nour@alexu.edu.eg (I.H.N.)

Table S1. Descriptive data for vegetative macromorphology, germination, and photosynthetic pigments traits of *Trianthema portulacastrum*.

Traits	Variable	Mean ± StDev	SE Mean	Min.	Q1	Median	Q3	Max.	IQR
Vegetative macromorphology	No. of secondary branches	8.514 ± 3.293	0.557	3	6	8	11	17	5
	No. of tertiary branches	5.371 ± 1.972	0.333	3	4	5	7	9	3
	No. of primary branch internodes	9.971 ± 3.129	0.529	5	7	10	13	15	6
	No. of secondary branch internodes	6.857 ± 2.415	0.408	3	5	7	8	12	3
	Max internode length of primary branch (cm)	8.414 ± 2.221	0.375	4.5	7	8	10	14.5	3
	Min internode length of primary branch (cm)	1.0886 ± 0.3902	0.0659	0.5	0.9	1	1	2.2	0.1
	Max internode length of secondary branch (cm)	6.349 ± 1.454	0.246	4	5.5	6	7	10.5	1.5
	Min internode length of secondary branch (cm)	0.82 ± 0.3454	0.0584	0.3	0.5	0.7	1	1.5	0.5
	Single leaf petiole length (cm)	1.5171 ± 0.4768	0.0806	0.5	1.2	1.5	1.7	3	0.5
	Single leaf blade length (cm)	3 ± 0.732	0.124	1.4	2.4	3	3.6	4.4	1.2
	Single leaf blade width (cm)	2.651 ± 0.825	0.139	1.2	2	2.7	3	4.5	1
	Single leaf L/W ratio	1.1642 ± 0.1716	0.029	0.9048	1	1.1429	1.2857	1.5333	0.2857
	Single leaf area (cm^2)	5.653 ± 2.91	0.492	1.12	3.84	5.667	6.827	13.2	2.987
	Leaf petiole length (cm)	0.5441 ± 0.2619	0.0449	0.2	0.3	0.5	0.7	1.3	0.4
	Leaf blade length (cm)	1.2714 ± 0.3586	0.0606	0.9	1	1.2	1.5	2.3	0.5
	Leaf blade width (cm)	0.9171 ± 0.312	0.0527	0.5	0.8	0.8	1	1.8	0.2
	Leaf L/W ratio	1.4221 ± 0.2223	0.0376	1.0556	1.25	1.4286	1.5	2	0.25
	Leaf area (cm^2)	0.8425 ± 0.5827	0.0985	0.3	0.5333	0.64	0.84	2.76	0.3067
Germination	Radical length (cm)	2.498 ± 0.791	0.134	1.3	1.733	2.51	3	4.433	1.267
	Plumule length (cm)	2.6723 ± 0.39	0.066	1.733	2.43	2.7	2.933	3.533	0.503
	Prophyllus length (mm)	0.37905 ± 0.0499	0.008	0.266	0.333	0.366	0.4	0.466	0.066
Photosynthetic Pigments	Chl a (mg/g Fwt)	5.005 ± 1.361	0.23	2.61	4.15	4.94	5.97	8.06	1.82
	Chl b (mg/g Fwt)	1.5841 ± 0.5119	0.0865	0.68	1.3	1.49	1.82	2.68	0.52
	Chl a + Chl b (mg/g Fwt)	6.589 ± 1.801	0.304	3.31	5.3	6.44	7.75	10.33	2.45
	Carotenoids (mg/g Fwt)	1.1456 ± 0.3379	0.0571	0.6385	0.9078	1.109	1.3726	1.8986	0.4648
	Chl/ Car (mg/g Fwt)	5.837 ± 0.826	0.14	4.505	5.229	5.741	6.42	8.094	1.191
	Chl a/b (mg/g Fwt)	3.2529 ± 0.518	0.0876	1.6978	2.9889	3.2748	3.6087	4.6923	0.6198

Table S2. Quantitative characteristics of Abaxial leaf and Adaxial leaf micromorphology of *Trianthema portulacastrum*.

	Variable	Mean ± SD	SE Mean	Minimum	Q1	Median	Q3	Maximum	IQR
Abaxial leaf	Stomatal Index (%)	28.88±6.26	1.4	16.67	25	30	33.33	37.5	8.33
	Stomatal complex length (opened) (μm)	21.404±7.146	0.964	11.698	17.441	19.56	20.989	42.906	3.548
	Stomatal complex width (opened) (μm)	7.128±2.256	0.304	4.297	5.587	6.644	7.789	13.149	2.202
	Stomatal complex L/W ratio (opened)	3.071±0.711	0.0959	1.7755	2.6781	2.9419	3.4285	5.8423	0.7504
	Stomatal pore length (μm)	13.36±6.527	0.88	6.575	9.046	11.382	13.764	32.059	4.718
	Stomatal pore width (μm)	2.299±1.328	0.179	0.562	1.141	2.107	3.175	7.081	2.034
	Stomatal pore L/W ratio	6.997±3.396	0.458	2.458	4.039	6.619	9.482	17.434	5.443
	Stomatal complex length (closed) (μm)	17.348±4.082	0.527	11.683	15.302	16.857	17.899	37.061	2.597
	Stomatal complex width (closed) (μm)	4.747±1.152	0.149	2.669	3.901	4.589	5.297	9.277	1.395
	Stomatal complex L/W ratio (closed)	3.765±0.831	0.107	2.531	3.084	3.68	4.232	6.431	1.149
	Epidermal cell length (μm)	44.79±20.83	3.74	19.84	31.29	40.55	47.01	101.49	15.72
	Epidermal cell width (μm)	36.4±25.38	4.56	11.35	23.59	27.73	43.01	121.59	19.42
	Epidermal cell L/W ratio	1.4032±0.4613	0.0829	0.3796	1.1229	1.3758	1.7004	2.3761	0.5775
	Epidermal cell area (μm^2)	1672±2008	361	202	676	936	1413	9941	737
	Subsidiary cell length (μm)	31.34±9.89	1.36	15.77	23.59	31.89	36.74	64.95	13.16
	Subsidiary cell width (μm)	15.303±7.119	0.978	6.997	11.662	12.91	16.07	47.334	4.408
	Subsidiary cell L/W ratio	2.2162±0.6236	0.0857	0.4984	1.9287	2.1788	2.6916	3.5469	0.7629
	Subsidiary cell area (μm^2)	455.6±272.7	37.5	128.4	293.8	416.4	520.1	1482.2	226.3
Adaxial leaf	Stomatal Index (%)	20.86±11.13	2.37	0	19.17	25	28.57	37.5	9.4
	Stomatal complex length (opened) (μm)	21.497±5.91	0.739	12.2	18.31	20.324	23.054	42.162	4.744
	Stomatal complex width (opened) (μm)	7.018±2.498	0.312	4.083	5.261	6.378	8.385	17.408	3.125
	Stomatal complex L/W ratio (opened)	3.2068±0.7929	0.0991	1.9385	2.642	3.1289	3.5278	5.9854	0.8858
	Stomatal pore length (μm)	13.766±5.592	0.699	4.116	10.378	12.982	16.555	31.077	6.176
	Stomatal pore width (μm)	2.452±1.393	0.174	0.792	1.453	1.965	3.026	7.081	1.573
	Stomatal pore L/W ratio	6.628±3.355	0.419	2.998	4.152	5.646	8.375	18.441	4.223
	Stomatal complex length (closed) (μm)	18.88±5.465	0.864	12.418	15.738	18.198	20.484	42.306	4.746
	Stomatal complex width (closed) (μm)	4.806±1.251	0.198	2.427	4.037	4.698	5.457	7.688	1.42
	Stomatal complex L/W ratio (closed)	4.128±1.372	0.217	2.475	3.194	3.754	4.771	8.162	1.577

Epidermal cell length (μm)	49.78±21.06	3.67	22.15	31.62	45.09	63.25	101.95	31.63
Epidermal cell width (μm)	46.39±27.99	4.87	17.71	25.52	37.5	58.49	144.83	32.97
Epidermal cell L/W ratio	1.2129±0.4011	0.0698	0.4443	0.8407	1.2527	1.5381	2.1334	0.6973
Epidermal cell area (μm^2)	2289±2034	354	454	757	1589	2757	7818	2000
Subsidiary cell length (μm)	35.66±11.77	1.68	20.47	26.62	31.64	45	66.98	18.37
Subsidiary cell width (μm)	16.743±6.924	0.989	7.827	11.675	14.985	19.84	41.346	8.165
Subsidiary cell L/W ratio	2.31±0.772	0.11	0.767	1.779	2.33	2.714	4.564	0.934
Subsidiary cell area (μm^2)	590.8±383.8	54.8	233.5	337.9	415.2	764.1	1569.3	426.2

Table S3. Qualitative characteristics of Abaxial leaf and Adaxial leaf micromorphology of *Trianthema portulacastrum*.

	Characters/ specimen No	2	4	7	12	21	25	28	33	35
Abaxial	**Epidermal cell outline**	Isodiametric hexagonal to polygonal	Isodiametric hexagonal to polygonal	Isodiametric hexagonal to polygonal	Isodiamertic tetragonal to polygonal	Isodiamertic pentagonal to hexagonal	Isodiamertic hexagonal to polygonal	Isodiamertic pentagonal to hexagonal	Isodiamertic hexagonal to polygonal	Isodiamertic hexagonal to polygonal
	**Anticlinal wall**	Irregularly curved	Irregularly curved	Irregularly curved	Straight	Irregularly curved	Irregularly curved	Straight	Irregularly curved	Irregularly curved
	**Curvature of outer periclinal wall**	Flat	Convex	Flat	Flat	Convex	Convex	Convex	Convex	Convex
	**Fine relief of the cell wall**	Slightly striate	Striate	Smooth	Smooth	Slightly striate	Slightly striate	Slightly striate	Slightly striate	Slightly striate
	**Guard cell surface**	Smooth	Very thin platelets	Smooth	Smooth	Smooth	Very thin platelets	Smooth	Smooth	Smooth
	**Pore shape**	Elliptic	Elliptic	Elliptic	Elliptic	Linear	Elliptic	Elliptic	Linear	Linear
Adaxial	**Epidermal cell outline**	Isodiametric hexagonal to polygonal	Isodiametric polygonal	Isodiametric hexagonal to polygonal	Isodiamertic tetragonal to hexagonal	Isodiamertic pentagonal to hexagonal	Isodiamertic pentagonal to hexagonal	Isodiamertic pentagonal to hexagonal	Isodiamertic hexagonal to polygonal	Isodiamertic hexagonal to polygonal
	**Anticlinal wall**	Irregularly curved	Irregularly curved	Irregularly curved	Straight	Irregularly curved	Irregularly curved	Straight	Irregularly curved	Irregularly curved
	**Curvature of outer periclinal wall**	Flat	Convex	Flat	Convex	convex	convex	Convex	Convex	Convex
	**Fine relief of the cell wall**	Slightly striate	Striate	Smooth	Smooth	Smooth	Smooth	Slightly striate	Slightly striate	Smooth
	**Guard cell surface**	Smooth	Very thin platelets	Smooth	Smooth	Smooth	Very thin platelets	Smooth	Smooth	Smooth
	**Pore shape**	Elliptic	Linear	Elliptic	Elliptic	Linear	Elliptic + Linear	Elliptic	Linear	Elliptic
	**Epicuticular secretion type**	Film-like	Irregular platelets with irregular margin	Film-like	Irregular platelets with irregular margin	Irregular platelets with irregular margin	Irregular platelets with irregular margin	Film-like	Film-like	Irregular platelets with irregular margin
	**Epicuticular secretions density**	N/A	Moderate	N/A	Sparse	Moderate	Moderate	N/A	N/A	Moderate

Table S4. Two-Way ANOVA analysis of Abaxial leaf and Adaxial leaf micromorphological characters of *Trianthema portulacastrum*.

Characters	Group-TE		Group-YB		Group-FS	
	Abaxial	Adaxial	Abaxial	Adaxial	Abaxial	Adaxial
Stomatal Index (%)	28.23 ± 8.24[a]	26.5 ± 7.42[a]	27.4 ± 5.43[a]	26.03 ± 5.07[a]	33.54 ± 3.07[a]	11.53 ± 12.43[a]
Stomatal complex length (opened) (µm)	20.039 ± 1.782[abc]	21.98 ± 5.2[ab]	24.75 ± 9.25[a]	23.82 ± 6.27[a]	17.908 ± 2.662[bc]	17.262 ± 2.814[c]
Stomatal complex width (opened) (µm)	6.441 ± 0.891[abc]	6.363 ± 3.487[c]	7.894 ± 2.877[ab]	8.247 ± 2.087[a]	6.513 ± 1.49[bc]	5.396 ± 0.676[c]
Stomatal complex L/W ratio (opened)	3.143 ± 0.341[ab]	3.844 ± 1.207[a]	3.238 ± 0.921[ab]	2.945 ± 0.593[b]	2.826 ± 0.455[b]	3.211 ± 0.457[ab]
Stomatal pore length (µm)	11.418 ± 2.303[abc]	13.72 ± 4.24[ab]	16.36 ± 8.44[a]	16.46 ± 5.79[a]	10.587 ± 2.591[bc]	9.267 ± 2.345[c]
Stomatal pore width (µm)	1.63 ± 0.884[b]	1.802 ± 0.714[ab]	2.475 ± 1.599[ab]	3.125 ± 1.607[a]	2.412 ± 1.058[ab]	1.764 ± 0.63[b]
Stomatal pore L/W ratio	8.8 ± 4.18[a]	8.46 ± 3.72[a]	7.726 ± 3.216[a]	6.425 ± 3.583[b]	5.182 ± 2.355[b]	5.721 ± 2.173[ab]
Stomatal complex length (closed) (µm)	16.573 ± 1.716[bc]	17.433 ± 3.372[bc]	18.98 ± 5.63[ab]	22.76 ± 7.92[a]	15.066 ± 1.547[c]	17.32 ± 3.44[abc]
Stomatal complex width (closed) (µm)	5.123 ± 0.714[ab]	4.296 ± 1.017[c]	4.59 ± 1.515[bc]	6.025 ± 0.852[a]	4.096 ± 0.535[bc]	4.529 ± 1.282[abc]
Stomatal complex L/W ratio (closed)	3.288 ± 0.533[b]	4.287 ± 1.355[a]	4.271 ± 0.864[a]	3.897 ± 1.69[ab]	3.742 ± 0.65[ab]	3.943 ± 0.793[ab]
Epidermal cell length (µm)	43.15 ± 19.7[ab]	37.64 ± 16.39[ab]	46.05 ± 24.26[ab]	58.48 ± 20.37[a]	44.27 ± 9.12[ab]	31.46 ± 3.72[b]
Epidermal cell width (µm)	31.48 ± 13.77[b]	32.8 ± 22.13[ab]	42.3 ± 32.75[ab]	56.72 ± 28.24[a]	26.32 ± 6.1[ab]	23.84 ± 7.34[b]
Epidermal cell L/W ratio	1.42 ± 0.344[a]	1.249 ± 0.288[a]	1.306 ± 0.502[a]	1.1399 ± 0.3979[a]	1.745 ± 0.515[a]	1.433 ± 0.481[a]
Epidermal cell area (μm^2)	1225 ± 1124[b]	1435 ± 1553[ab]	2159 ± 2583[ab]	2992 ± 2119[a]	950 ± 248[ab]	685 ± 259[b]
Subsidiary cell length (µm)	34.78 ± 6.01[ab]	39.98 ± 13.55[a]	29.81 ± 12.41[b]	33.93 ± 10.28[ab]	27.96 ± 8.19[b]	30.11 ± 7.1[ab]
Subsidiary cell width (µm)	15.38 ± 4.88[a]	17.1 ± 5.33[a]	14.17 ± 8.36[a]	18.72 ± 9.27[a]	17.77 ± 7.85[a]	13 ± 3.68[a]
Subsidiary cell L/W ratio	2.359 ± 0.478[a]	2.458 ± 0.882[a]	2.246 ± 0.599[a]	2.031 ± 0.588[a]	1.861 ± 0.837[a]	2.458 ± 0.74[a]
Subsidiary cell area (μm^2)	495.1 ± 186.6[ab]	677.9 ± 415[a]	438.7 ± 363.1[b]	637.8 ± 405.1[b]	415.5 ± 167[ab]	352.1 ± 132.2[ab]

Table S5. Quantitative characteristics of *Trianthema portulacastrum* seed micromorphology.

Variable	Position	Mean ± StDev	SE Mean	Minimum	Q1	Median	Q3	Maximum	IQR
Epidermal cell count	Lateral side (Center)	65.44 ± 6.25	2.08	57	61	64	69.5	78	8.5
	Lateral side (Edge)	* ± *	*	*	*	*	*	*	*
	Dorsal side	53.56 ± 6.19	2.06	41	50	54	58.5	61	8.5
	Ventral side	48.22 ± 10	3.33	26	43.5	49	56	59	12.5
Epidermal cell length (μm)	Lateral side (Center)	16.737 ± 3.045	0.364	10.15	14.512	16.52	18.535	27.02	4.023
	Lateral side (Edge)	27.687 ± 6.041	0.712	17.12	22.725	26.995	32.017	43.27	9.293
	Dorsal side	18.188 ± 3.97	0.468	9.82	14.732	17.715	21.08	28.85	6.347
	Ventral side	16.584 ± 3.387	0.399	9.6	14.76	16.52	18.8	26.19	4.04
Epidermal cell width (μm)	Lateral side (Center)	13.881 ± 2.478	0.296	8.54	12.27	13.45	15.563	19.17	3.293
	Lateral side (Edge)	15.353 ± 3.17	0.374	10.27	13.06	15.425	17.26	24.29	4.2
	Dorsal side	13.052 ± 2.544	0.304	7.97	11.23	12.765	14.9	18.49	3.67
	Ventral side	11.729 ± 2.726	0.321	6.31	9.813	12.04	13.503	20.19	3.69
Epidermal cell L/W ratio	Lateral side (Center)	1.2244 ± 0.2215	0.0265	0.79	1.0475	1.205	1.3725	1.75	0.325
	Lateral side (Edge)	1.8519 ± 0.4563	0.0538	1.07	1.5325	1.78	2.11	3.03	0.5775
	Dorsal side	1.4181 ± 0.3118	0.0373	0.91	1.2075	1.345	1.6	2.64	0.3925
	Ventral side	1.4507 ± 0.2934	0.0346	0.94	1.2225	1.415	1.6175	2.64	0.395
Epidermal cell area (μm^2)	Lateral side (Center)	193.7 ± 58.12	6.95	68.97	152.22	187.16	228.43	337.57	76.21
	Lateral side (Edge)	371.6 ± 125.8	14.8	158.9	274.9	349.7	456.8	741.5	181.8
	Dorsal side	199.01 ± 70.73	8.34	65.65	150.57	183.21	252.03	385.9	101.46
	Ventral side	157.21 ± 55.82	6.58	54.02	116.93	152.83	190.95	292.66	74.02

Table S6. Qualitative characteristics of *Trianthema portulacastrum* seed micromorphology for the lateral side.

Characters/ samples	Lateral side									
	Center position					Edge position				
	Epidermal cell outline	Anticlinal wall	Relief of cell boundary	Periclinal wall protrusion shape	Fine relief of the cell wall	Epidermal cell outline	Anticlinal wall	Relief of cell boundary	Periclinal wall protrusion shape	Fine relief of the cell wall
**2**	Isodiametric tetragonal to heptagonal	Straight with curved angles	Highly raised	Dome-like	Striate	Oblong	Straight	Moderately raised	Wedge-like	Slightly striate
**4**	Isodiametric pentagonal to heptagonal	Straight with curved angles	Highly raised	Wedge-like	Slightly striate	Isodiametric hexagonal to heptagonal	Straight with curved angles	Moderately raised	Dome-like	Striate
**7**	Rounded	Straight	Moderately raised	Wedge-like	Striate	Isodiametric pentagonal to heptagonal	Straight with curved angles	Moderately raised	Dome-like	Slightly striate
**12**	Isodiametric tetragonal to heptagonal	Straight with acute angles	Highly raised	Dome-like	Striate	Isodiametric pentagonal to hexagonal	Straight with curved angles	Moderately raised	Dome-like	Striate
**21**	Isodiametric tetragonal to heptagonal	Straight with curved angles	Highly raised	Wedge-like	Striate	Oblong to isodiametric tetragonal to hexagonal	Straight with curved angles	Moderately raised	Wedge-like	Slightly striate
**25**	Isodiametric pentagonal to heptagonal	Straight with acute angles	Highly raised	Wedge-like	Striate	Oblong to isodiametric pentagonal to heptagonal	Straight with curved angles	Highly raised	Dome-like	Slightly striate
**28**	Isodiametric pentagonal to octagonal cells	Straight with curved angles	Highly raised	Dome-like	Slightly striate	Oblong to isodiametric pentagonal to hexagonal	Straight with curved angles	Moderately raised	Wedge-like	Slightly striate
**33**	Isodiametric tetragonal to hexagonal	Straight with acute angles	Highly raised	Wedge-like	Striate	Oblong	Straight	Highly raised	Dome-like	Striate
**35**	Isodiametric tetragonal to heptagonal	Straight with curved angles	Highly raised	Dome-like	Slightly striate	Oblong	Straight	Highly raised	Wedge-like	Striate

Table S7. Qualitative characteristics of *Trianthema portulacastrum* seed micromorphology for the dorsal and ventral sides.

Characters /samples	Dorsal side (Center position)					Ventral side								
							Center position					Base position		
	Epidermal cell outline	Anticlinal wall	Relief of cell boundary	Periclinal wall protrusion shape	Fine relief of the cell wall	Hilum position	Epidermal cell outline	Anticlinal wall	Relief of cell boundary	Periclinal wall protrusion shape	Fine relief of the cell wall	Epidermal cell outline	Anticlinal wall	Relief of cell boundary
**2**	Rounded	Straight	Highly raised	Dome-like	Striate	Basal	Rounded	Straight	Highly raised	Dome-like	Striate	Rounded	Straight	Highly raised
**4**	Isodiametric tetragonal to hexagonal	Straight with curved angles	Highly raised	Dome-like	Striate	Basal	Rounded	Straight	Highly raised	Dome-like	Slightly striate	Rounded	Straight	Moderately raised
**7**	Isodiametric tetragonal to hexagonal	Straight with curved angles	Moderately raised	Papillose	Slightly striate	Basal	Isodiametric pentagonal to hexagonal	Straight with curved angles	Moderately raised	Papillose	Slightly striate	Isodiametric pentagonal to hexagonal	Straight with curved angles	Highly raised
**12**	Rounded	Straight	Highly raised	Dome-like	Striate	Sub-basal	Isodiametric pentagonal to hexagonal	Straight with curved angles	Moderately raised	Dome-like	Striate	Isodiametric pentagonal to hexagonal	Straight with curved angles	Highly raised
**21**	Rounded	Straight	Moderately raised	Dome-like	Slightly striate	Sub-basal	Rounded	Straight	Moderately raised	Dome-like	Slightly striate	Rounded	Straight	Highly raised
**25**	Isodiametric pentagonal to hexagonal	Straight with curved angles	Moderately raised	Dome-like or papillose	Striate	Basal	Rounded	Straight	Moderately raised	Dome-like	Slightly striate	Isodiametric pentagonal to hexagonal	Straight with curved angles	Highly raised
**28**	Rounded	Straight	Highly raised	Dome-like	Striate	Basal	Rounded	Straight	Highly raised	Dome-like	Striate	Rounded	Straight	Highly raised
**33**	Isodiametric pentagonal to hexagonal	Straight with curved angles	Highly raised	Dome-like	Striate	Sub-basal	Isodiametric tetragonal to hexagonal	Straight with curved angles	Highly raised	Dome-like	Striate	Rounded	Straight	Highly raised
**35**	Isodiametric tetrtagonal to octagonal	Straight with acute angles	Moderately raised	papillose	Slightly striate	Basal	Rounded	Straight	Highly raised	Dome-like	Slightly striate	Rounded	Straight	Moderately raised

Table S8. Two-Way ANOVA analysis of *Trianthema portulacastrum* seed micromorphology.

Groups	Position	Epidermal cell count	Epidermal cell length (μm)	Epidermal cell width (μm)	Epidermal cell L/W ratio	Epidermal cell area (μm^2)
**Group-TE**	Lateral side (Center)	65.33 ± 3.21^{ab}	17.075 ± 3.081^{b}	13.603 ± 2.198^{bc}	1.2639 ± 0.1964^{de}	196.8 ± 59.1^{c}
	Lateral side (Edge)	* ± *	27.53 ± 6.61^{a}	15.073 ± 2.763^{ab}	1.8512 ± 0.4284^{ab}	370.3 ± 151.7^{a}
	Dorsal	54 ± 5.29^{bc}	17.268 ± 3.622^{b}	13.189 ± 2.545^{bcd}	1.3237 ± 0.2308^{cde}	188.5 ± 76.6^{cd}
	Ventral	47 ± 18.2^{bc}	16.45 ± 2.54^{b}	12.415 ± 2.961^{cd}	1.3808 ± 0.3164^{cde}	168.9 ± 53.8^{cd}
**Group-YB**	Lateral side (Center)	61.25 ± 3.77^{bc}	16.953 ± 3.411^{b}	14.3 ± 2.578^{abc}	1.2071 ± 0.2513^{e}	203.3 ± 58.3^{c}
	Lateral side (Edge)	* ± *	28.97 ± 6.46^{a}	16.436 ± 3.372^{a}	1.8109 ± 0.4668^{ab}	389.5 ± 124.8^{a}
	Dorsal	55.5 ± 4.65^{bc}	17.955 ± 3.632^{b}	12.467 ± 2.218^{cd}	1.4597 ± 0.3474^{cd}	186.65 ± 54.27^{c}
	Ventral	46 ± 2.94^{c}	16.537 ± 4.141^{b}	10.993 ± 2.54^{d}	1.5237 ± 0.2886^{bcd}	143 ± 58.7^{d}
**Group-FS**	Lateral side (Center)	74 ± 5.66^{a}	15.834 ± 2.079^{b}	13.469 ± 2.686^{bcd}	1.2013 ± 0.1981^{de}	170.6 ± 53.4^{cd}
	Lateral side (Edge)	* ± *	25.369 ± 3.19^{a}	13.607 ± 2.548^{abc}	1.935 ± 0.493^{ab}	337.8 ± 74.4^{ab}
	Dorsal	49 ± 11.31^{bc}	20.03 ± 4.7^{b}	14.041 ± 2.978^{abc}	1.4833 ± 0.3306^{bcd}	239.5 ± 79.7^{bc}
	Ventral	54.5 ± 2.12^{bc}	16.877 ± 2.973^{b}	12.174 ± 2.506^{cd}	1.4094 ± 0.2462^{cde}	168.1 ± 49.2^{cd}

Table S9. Voucher information of 35 samples representing *Trianthema portulacastrum* collected from Fayoum Depression (FD) in Egypt.

Sample Code	Locality	Longitude	Latitude	Elevation (m)
**T1 E**	Daniel, Etsa	30°45'6.9" E	29°7'0.9" N	17
**T2 E**	Al Gharq, Qebli, Etsa	30°42'18.3" E	29°7'51" N	8
**T3 E**	Al Gharq−Fayoum Road, Izbat Al Daw, Etsa	30°45'50.1" E	29°12'44.4" N	10
**T4 E**	Al Gaafra, Minya Al Hayt, Etsa	30°45'29.5" E	29°13'32.8" N	12
**T5 E**	Minya Al Hayt, Abu Jandir Road, Al Awfi, Etsa	30°42'3.5" E	29°14'8.6" N	5
**T6 E**	Madinat Al Fayoum, Ibshawy Road, Gerdo, Etsa	30°43'12.6" E	29°18'01.8" N	15
**T7 F**	Hawaret Al Maqtaa, Ezbet Ali Farag, Al Hadqa, Al Fayoum	30°53'32.3" E	29°15'8.2" N	23
**T8 F**	Ezbet Ali Farag, Al Hadqa, Al Hadeqah, Al Fayoum	30°50'42.5" E	29°16'2.9" N	22
**T9 F**	Abgig, Al Fayoum	30°48'57.67" E	29°16'56.351" N	16
**T10 F**	Senofar, Al Fayoum	30°52'41.2" E	29°17'12.6" N	27
**T11 F**	Madinat Al Fayoum, Ibshawy Gate on Ring Road, Abgig−Al Fayoum	30°48'14.153" E	29°17'55.397" N	19
**T12 F**	Qesm Al Fayoum, Al Fayoum	30°49'03.1" E	29°18'01.0" N	20
**T13 F**	Kofour Al Nil, Al Fayoum	30°53'23.14" E	29°18' 46.63" N	20
**T14 F**	Al Eelam, Al Fayoum	30°52'07.5" E	29°19'38.2" N	20
**T15 F**	El-Mandara, Al Fayoum	30°48'34.8" E	29°19'54.9" N	20
**T16 F**	Al Edwah, Al Fayoum	30°55'52" E	29°19'58.7" N	18
**T17 F**	Zawyet Al Kerdaseya, Bani Saleh, Al Fayoum	30°48'18.9" E	29°21'18.8" N	21
**T18 S**	Behmo, Senouris	30°50'51.4" E	29°22'27.1" N	16
**T19 S**	Madinat Al Fayoum, Kafr Mahfouz Road, Matartares, Senouris	30°54'17.1" E	29°22'46.1" N	14

**T20 S**	Madinat Al Fayoum, Tersa Road, Naqalifah, Senouris	30°49'31.9" E	29°24'39.8" N	-2
**T21 S**	Ezbet Mohammed Mahfouz, Senouris	30°53'03.4" E	29°26'08.3" N	-13
**T22 T**	Qasr Rashwan, Tamia	30°55'22.5" E	29°27'30.4" N	-12
**T23 T**	Madinet Tamia, Tamia	30°56'28.1" E	29°29'02.2" N	-13
**T24 T**	Madinet Tamia, Tamia	30°58'41.9" E	29°29'25.5" N	-8
**T25 T**	Monshaat Doctor El-Gammal, Tamia	31°03'21.3" E	29°30'32.1" N	8
**T26 T**	Kafr Al Maslat, Tamia, Fanous, Tameyah	30°58'55.9" E	29°32'52.5" N	12
**T27 B**	Madinat Al Fayoum, Tohbar Road, Al Agameyin, Ibshawy	30°42'58.6" E	29°19'48.2" N	14
**T28 B**	Al Agameyin–Ibshawy Road, Zaid, Ibshawy	30°41'26.0" E	29°20'57.9" N	17
**T29 B**	Qasr Bayad, Ibshawy	30°44'12.1" E	29°21'30.2" N	18
**T30 Y**	Al Hamouli, Yousef El-Seddik	30°37'15.4" E	29°17'10" N	0
**T31 Y**	Al Nazlah, Yousef El-Seddik	30°38'14.4" E	29°17'59.8" N	-3
**T32 Y**	Qasr Al Gabali, Yousef El-Seddik	30°37'33.2" E	29°19'57.8" N	6
**T33 Y**	Al Shawashna, Ezbet Gabal Saed, Qasr Al Gabali, Yousef El-Seddik	30°38'38.4" E	29°21'04.9" N	-46
**T34 Y**	Izbat Burish, Qarun Lake Touristic Road, Al Mashrak, Yousef El-Seddik	30°33'30.6" E	29°24'40.0" N	-33
**T35 Y**	Kahk, Yousef El-Seddik	30°38'39.4" E	29°26'02.0" N	-42
